# Tailoring Magnetic Properties and Power Loss in Low-Temperature Sintered NiCuZn Ferrites with BMLS-CaTiO_3_/BaTiO_3_ Composite Additives

**DOI:** 10.3390/ma18225202

**Published:** 2025-11-17

**Authors:** Chuan Chen, Zhengfeng Cao, Lei Cui, Fangyuan Chang, Yan Xiao, Lining Wu, Xiangyu Ge

**Affiliations:** 1 State Key Laboratory of Advanced Power Transmission Technology, China Electric Power Research Institute Co., Ltd., Beijing 102209, China; chenchuan1@epri.sgcc.com.cn (C.C.); cuilei@geiri.sgcc.com.cn (L.C.); changfangyuan@epri.sgcc.com.cn (F.C.); xiaoyan@epri.sgcc.com.cn (Y.X.); 2 School of Integrated Circuits, Chongqing University of Posts and Telecommunications, Chongqing 400065, China; 3 School of Energy Power and Mechanical Engineering, North China Electric Power University, Beijing 102206, China; 23102059@ncepu.edu.cn; 4 School of Mechanical Engineering, Beijing Institute of Technology, Beijing 100081, China

**Keywords:** NiCuZn, BMLS, CaTiO_3_, BaTiO_3_, magnetic properties, power loss

## Abstract

In this study, NiCuZn ferrites were prepared via a solid-phase method at a reduced sintering temperature of 925 °C. A composite additive system was developed by incorporating Bi_2_O_3_-MgO-Li_2_CO_3_-SiO_2_ (BMLS) glass with varying amounts of either CaTiO_3_ or BaTiO_3_, aiming to modify the magnetic performance and power loss behavior of the ferrites. The effects of BMLS-CaTiO_3_ (Group A) and BMLS–BaTiO_3_ (Group B) on the crystalline structure, density, electrical resistivity, microstructure, magnetic properties, as well as power loss were systematically investigated, and the underlying mechanisms were thoroughly discussed. Magnetic characterization revealed that the addition of BMLS-CaTiO_3_/BaTiO_3_ decreased the saturation magnetization (*Ms*) and permeability, primarily due to the magnetic dilution, as well as a reduction in density and grain size. However, the intrinsic coercivity increased, which can be attributed to the pinning of magnetic domain walls by the glass phase located at the grain boundaries and the reduction in crystallite size. The resistivity increased first and then decreased with the increasing content of CaTiO_3_/BaTiO_3_. When x = 0.1, samples in Group A and Group B reached the maximum values of 473.16 MΩ·m and 453.12 MΩ·m, respectively. Nonetheless, with an additive content of 0.05 wt%, samples in Group A and Group B still exhibited comparatively high *Ms* values of 53.93 emu/g and 54.65 emu/g, as well as high permeability values of 636.95 and 651.55, respectively. Meanwhile, power loss measurements indicated a notable reduction in total power loss across various frequencies and magnetic induction levels for both groups, with losses decreasing by more than 35%. Further analysis attributed these changes to enhanced grain uniformity, improved densification, and increased resistivity resulting from the incorporation of BMLS-CaTiO_3_/BaTiO_3_.

## 1. Introduction

In recent decades, NiCuZn ferrite has garnered a lot of interest in both academia and industry because of its numerous significant advantages, including high magnetic permeability, high frequency, low power loss, a simple preparation method, and so forth. NiCuZn ferrite has become a fundamental material in the field of electronic information [1,2,3,4]. Electronic devices are advancing rapidly with trends toward greater integration, miniaturization, and multifunctionality. In this context, low-temperature co-fired ceramic (LTCC) technology has emerged as a key enabling method to meet these objectives [5,6,7,8]. A fundamental requirement of LTCC processing is the co-firing of ferrite materials alongside silver electrodes, which necessitates that the sintering temperature remains below the melting point of silver (961 °C). However, conventional NiCuZn ferrite is unsuitable for LTCC applications due to its high sintering temperature, which exceeds 1400 °C [5,6,7,8]. Meanwhile, low sintering temperatures can also adversely affect ferrite performance through reduced homogeneity and compactness, increasing porosity and inadequate grain growth, which can lead to a dramatic decrease in the magnetic properties of NiCuZn ferrites [9,10]. Therefore, obtaining low-temperature sintered magnetic materials with favorable magnetic properties has been a focus of research over the past two decades.

Previous studies have demonstrated that incorporating low melting point oxides as agents can effectively reduce the sintering temperature of NiCuZn ferrites. Additives such as Bi_2_O_3_, V_2_O_5_, Co_2_O_3_, Nb_2_O_5_, and La_2_O_5_ facilitate the formation of a liquid phase during sintering, which significantly enhances atomic diffusion and promotes the development of a dense microstructure in the ferrite material [9,10,11,12,13,14]. For instance, Ji et al. investigated the influence of Bi_2_O_3_ on the electromagnetic properties of NiCuZn ferrites sintered at 925 °C. Their results indicated that the addition of 0.3 wt% Bi_2_O_3_ promotes grain growth and leads to a change in electromagnetic performance [10]. Shen et al. investigated the influence of Co_2_O_3_–Bi_2_O_3_ on the microstructure and electromagnetic properties of NiCuZn ferrites sintered at 925 °C. Their results demonstrated that the samples possessed outstanding magnetic performance [12]. Similarly, Huo et al. introduced a composite additive of La_2_O_3_–Bi_2_O_3_ into NiCuZn ferrites, and found that specimens sintered at 900 °C showed superior magnetic properties along with reduced power loss [14].

The power loss characteristic of NiCuZn ferrites represents a critical performance parameter, garnering considerable academic and industrial interest in light of the advancing miniaturization and integration of modern electronic components. NiCuZn ferrites with a low power loss can dramatically reduce energy loss and enhance the electronic device efficiency [14,15,16,17,18]. Related studies showed a strong correlation between the power loss and resistivity [14]. High resistivity could make a contribution to decreasing the eddy current loss, thereby reducing the total power loss [14,16,19]. It is possible to increase the resistivity of NiCuZn ferrites by adding high resistivity and high melting point additives [14]. During the process of sintering NiCuZn ferrites, these high resistivity and high melting point additives could be enriched in the grain boundary and thus increase the resistivity [20,21,22].

**Figure 1:**
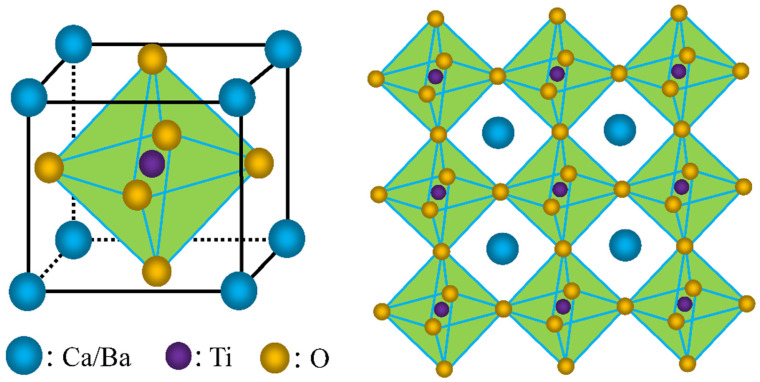
Crystal structure of CaTiO_3_ and BaTiO_3_.

According to the principles of glass formation, the Bi_2_O_3_-MgO-Li_2_CO_3_-SiO_2_ (BMLS) glass system exhibits a low softening temperature, making it a potential sintering aid for reducing the sintering temperature [6,7]. Meanwhile, CaTiO_3_ and BaTiO_3_ are perovskite-type compounds known for their high electrical resistivity and high melting points, which are desirable for suppressing power loss in NiCuZn ferrites. Figure 1 gives the crystal structure diagram of CaTiO_3_ and BaTiO_3_, which belong to the cubic crystal system and are composed of titanium/oxygen octahedra and barium/calcium/oxygen dodecahedra. Currently, limited studies have been reported on the combined effects of BMLS glass with either CaTiO_3_ or BaTiO_3_ as composite additives on the magnetic properties and power loss behavior of NiCuZn ferrites. Moreover, the underlying mechanism responsible for reducing power loss remains insufficiently explored.

In this study, BMLS glass was initially synthesized and then incorporated with CaTiO_3_ or BaTiO_3_ to form composite additives aimed at modulating the magnetic performance and power loss properties of NiCuZn ferrites. Some studies have reported that the *Ms* of NiCuZn ferrites ranges from 40 to 78 emu/g, while the intrinsic coercivity falls within 8–32 Oe [23,24,25]. In this work, compared with some reported samples, the addition of 3 wt% BMLS resulted in an increase in *Ms* and a decrease in intrinsic coercivity. This change is attributed to the liquid phase formed by BMLS during sintering, which facilitates densification through enhanced ion dissolution and mass transport [17,18]. Therefore, the content of BMLS glass was fixed at 3.0 wt%. The concentrations of CaTiO_3_ or BaTiO_3_ were varied at 0.02 wt%, 0.05 wt%, 0.08 wt%, 0.10 wt%, and 0.15 wt%. Ni_0.22_Cu_0.18_Zn_0.6_Fe_1.98_O_4_ ferrites were prepared via the solid-state reaction method. The impacts of the BMLS–CaTiO_3_/BaTiO_3_ composites on the crystal structure, bulk density, electrical resistivity, microstructure, magnetic properties, and power loss characteristics of the ferrites were systematically investigated. Additionally, the relevant mechanisms were discussed in detail.

## 2. Materials and Methods

### 2.1. Materials

High-purity raw materials, including NiO, CuO, ZnO, Bi_2_O_3_, MgO, Li_2_CO_3_, SiO_2_ (all ≥99.9%), Fe_2_O_3_, CaTiO_3_, BaTiO_3_ (all ≥99.5%), and polyvinyl acetate (analytical reagent grade), were sourced from Sinopharm Chemical Reagent Co., Ltd. (Shanghai, China) and used in the experiments. Table 1 gives the typical properties of CaTiO_3_ and BaTiO_3_.

### 2.2. Fabrication of BMLS Glass

BMLS glass was synthesized using Bi_2_O_3_, MgO, Li_2_CO_3_, and SiO_2_ as raw materials in a specific mass ratio. After precise weighing and thorough mixing, the mixture was melted at 1500 °C for 4 h. It was then cooled naturally to room temperature. The resulting solid was pulverized and homogenized using a ball mill (Hunan Focucy Experimental Instrument Co., LTD, Changsha, China). Finally, the powdered material was annealed at 80 °C for 2 h to obtain the final BMLS glass product.

### 2.3. Fabrication of NiCuZn with Added BMLS-CaTiO_3_/BaTiO_3_ Composites

The composites with a formula of (97 − x) wt% NiCuZnFe_1.98_O_4_ − 3 wt% BMLS glass − x wt% MTiO_3_ (M = Ca or Ba) were synthesized via a solid-state reaction route. Initially, the raw materials were weighed and mixed with deionized water using a ball mill operated at 250 rpm for 24 h. The resulting mixture was dried and subsequently calcined at 800 °C for 3 h. Then, 3.0 wt% BMLS along with different amounts of CaTiO_3_ or BaTiO_3_ (0.00 wt%, 0.02 wt%, 0.05 wt%, 0.08 wt%, 0.10 wt%, and 0.15 wt%) were added to the calcined powder, followed by another 24 h milling step at 250 rpm. After drying, the powder was granulated using 10 wt% polyvinyl acetate (PVA) as a binder and then pressed into toroidal shapes under a pressure of 8 MPa. Finally, the samples were sintered at 925 °C for 3 h to obtain the NiCuZn ferrites containing BMLS-CaTiO_3_/BaTiO_3_. The prepared samples were categorized into two groups: Group A corresponded to BMLS-CaTiO_3_, and Group B to BMLS-BaTiO_3_.

### 2.4. Characterization

An X-ray diffractor XRD (Miniflex 600, Rigaku Corporation, Tokyo, Japan) with a copper tube (Kα, λ = 1.54 Å) was employed to identify the crystalline phase of the as-prepared NiCuZn samples. A scanning electron microscope SEM (FEI Quanta 400FEG, Thermo Fisher Scientific Inc., Waltham, MA, USA) operating at an accelerating voltage of 10 kV was employed to obtain the microstructure of the samples. An energy dispersive X-ray spectroscopy (EDS, Bruker, Bruker Corporation, Billerica, MA, USA) was employed to examine the chemical compositions of as-prepared NiCuZn ferrites under 10 kv accelerating voltage with 7.5 nm beam current. Based on Archimedes method, the bulk density of samples was measured in distilled water. A TH2683A (ChangZhou TongHui Electronic Co., Ltd, Changzhou, China) resistivity tester was used to measure the electrical resistivity of the specimens. The quality factor (Q-factor) and complex magnetic permeability were characterized using an Agilent E4991B RF (Keysight Technologies Inc., Santa Rosa, CA, USA) impedance analyzer. Magnetic hysteresis loops, intrinsic coercivity (*Hc*), and saturation magnetization (*Ms*) were acquired via a vibrating sample magnetometer (VSM, Lake Shore Cryotronics, Ltd, Westerville, OH, USA). The power loss (*Pcv*) of the ferrite samples was measured with an Iwatsu B-H analyzer SY8232 (Iwatsu Electric Co., Ltd., Tokyo, Japan).

## 3. Results and Discussion

**Figure 2:**
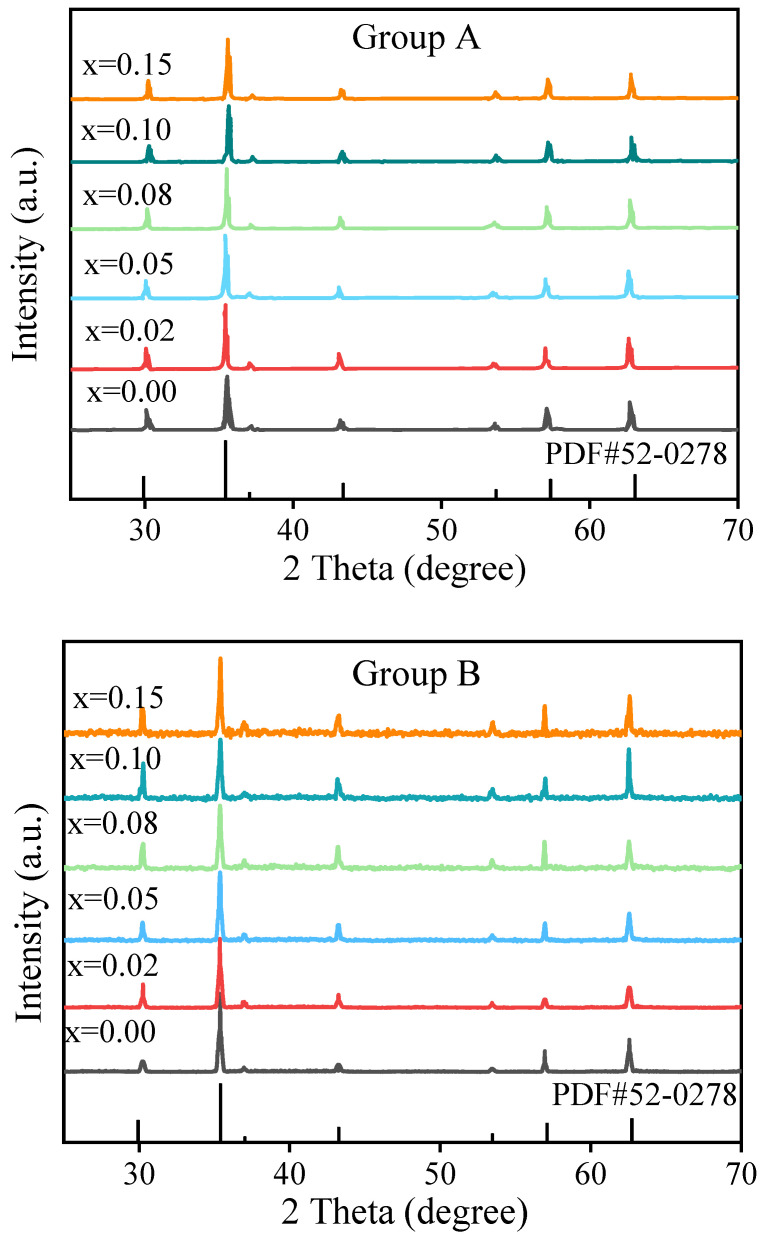
XRD patterns of NiCuZn ferrites with composite additives. The composition follows the formula: (97 − x) wt% NiCuZnFe_1.98_O_4_ − 3 wt% BMLS glass − x wt% MTiO_3_ (M = Ca or Ba). All samples exhibit a single-phase cubic spinel structure, and no secondary phases are detected with varying x.

Figure 2 presents the XRD patterns of two series of NiCuZn samples containing 3.0 wt% BMLS combined with x wt% CaTiO_3_/BaTiO_3_ (where x = 0.00, 0.02, 0.05, 0.08, 0.10, and 0.15). As the concentrations of CaTiO_3_/BaTiO_3_ increase from 0.00 wt% to 0.15 wt%, both groups A and B maintain similar XRD profiles without significant changes. Diffraction peaks associated with the (220), (331), (222), (400), (422), (511), and (440) planes match well with the reference pattern JCPDS-52-278. The XRD pattern of the samples confirms the formation of a single-phase cubic spinel structure, with no detectable secondary phases, which is also consistent with previous studies [13,16]. Meanwhile, as listed in Table 2, the lattice constant (a) was obtained by using the Rietveld method, exhibiting a decrease with increasing Ca/BaTiO_3_ content for both groups. This observed lattice contraction can be rationalized by two primary mechanisms: (i) the incorporation of smaller cations into the spinel lattice, potentially substituting for larger Fe^3+^ ions; and (ii) the introduction of compressive lattice strain due to the presence of the secondary Ca/BaTiO_3_ and glass phases at the grain boundaries, which hinders lattice expansion [3,4]. Furthermore, the micro-strain (ε) and crystallite size (t) were estimated using the software program FullProf. As the additive content increases, the strain (ε) gradually increases, while the crystallite size (t) demonstrates a reduction with higher additive content. These trends indicate that the incorporation of CaTiO_3_ or BaTiO_3_ additives induces compressive lattice strain, likely due to ionic substitutions or the introduction of stress fields within the ferrite structure, leading to lattice contraction. The progressive decline in crystallite size suggests that the additives inhibit grain growth, possibly through segregation at grain boundaries or by altering nucleation dynamics [3,4].

**Figure 3:**
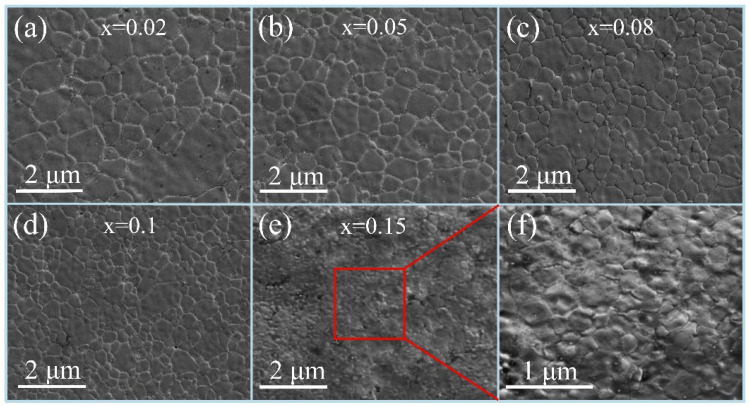
SEM images of NiCuZn ferrites with 3.0 wt% BMLS and varying amounts of CaTiO_3_ (x wt%). The composition parameter x represents the weight fraction of CaTiO_3_ in the composite additive system. The images show the evolution of grain size and uniformity as x increases: (**a**) x = 0.02, (**b**) x = 0.05, (**c**) x = 0.08, (**d**) x = 0.10, (**e**) x = 0.15. (**f**) is a local magnification of (**e**).

**Figure 4:**
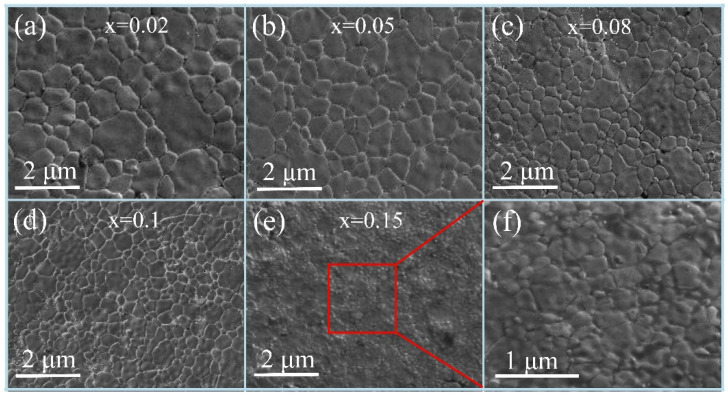
SEM images of NiCuZn ferrites with 3.0 wt% BMLS and varying amounts of BaTiO_3_ (x wt%). The composition parameter x represents the weight fraction of BaTiO_3_ in the composite additive system. The images show the evolution of grain size and uniformity as x increases: (**a**) x = 0.02, (**b**) x = 0.05, (**c**) x = 0.08, (**d**) x = 0.10, (**e**) x = 0.15. (**f**) is a local magnification of (**e**).

Figure 3 and Figure 4 depict the SEM images of the NiCuZn sample surfaces with added 3.0 wt% BMLS and different contents of CaTiO_3_/BaTiO_3_, respectively. Figure 3 and Figure 4 show that the addition of CaTiO_3_ or BaTiO_3_ resulted in similar changes in the grain size homogeneity and densification degree in the sintered NiCuZn ferrites. When the content of CaTiO_3_ or BaTiO_3_ increased from 0.02 wt% to 0.15 wt%, the grain size and densification degree of NiCuZn samples in group A and group B gradually decreased, and the grain size homogeneity initially improved and then deteriorated. CaTiO_3_ and BaTiO_3_ both have a relatively high melting point (over 1500 °C), which is much higher than the sintering temperature of 925 °C in this work. As a result, these high melting point additives were difficult to melt and existed in solid form during the sintering process, hindering material diffusion and grain growth. This resulted in a gradual reduction in the grain size of the NiCuZn samples, which is also consistent with the results shown in Table 2 [20,21,22].

Specifically, when x = 0.02, Figure 3a and Figure 4a showed that both large-sized and small-sized grains were simultaneously present in NiCuZn ferrite. The reason may be attributed to the relatively low content of CaTiO_3_ or BaTiO_3_, which exhibited a limited effect of crystal growth inhibition on grain growth. At a content of 0.05 wt% CaTiO_3_ or BaTiO_3_ (Figure 3b and Figure 4b), although the grain size decreased slightly, the grain size homogeneity was significantly improved compared to that in Figure 3a and Figure 4a, which indicated that the appropriate content of CaTiO_3_ or BaTiO_3_ was 0.05 wt%. When x = 0.08, 0.10, and 0.15, it can be observed that as the content of CaTiO_3_ or BaTiO_3_ further increased, the grain size and densification degree of the NiCuZn samples decreased rapidly. In particular, when x = 0.15, it was almost impossible to distinguish the grain morphology from Figure 3e and Figure 4e. The SEM images in Figure 3f and Figure 4f were locally enlarged images of Figure 3e and Figure 4e, respectively. From the local enlarged images, it could be seen that the grain size of the NiCuZn samples in group A and group B both became very small, and the grain boundaries also became very blurred. This is mainly due to the excessive addition of CaTiO_3_ or BaTiO_3_, which utilizes the effect of crystal growth inhibition to make the diffusion of the liquid-phase material difficult during the sintering process, resulting in the formation of small-sized grains, blurred grain boundaries, and decreased densification degree [14]. The microstructure characterization indicates that the addition of CaTiO_3_ or BaTiO_3_ significantly changes the grain size, homogeneity, and densification degree of the NiCuZn ferrites. When the content of CaTiO_3_ or BaTiO_3_ is 0.05 wt%, the NiCuZn samples in group A and group B both obtained relatively uniform grain size and dense microstructure.

Here, a more detailed discussion on the interaction between the BMLS glass and the CaTiO_3_/BaTiO_3_ phases during sintering is warranted to elucidate the microstructural evolution. Based on the XRD results (Figure 2), which show no evidence of secondary crystalline phases beyond the spinel structures, it is inferred that the interaction is likely dominated by physical processes. Referring to the previous studies [5,6,13], the low softening point of the BMLS glass contributes to the formation of a liquid phase at the sintering temperature. This liquid phase is expected to wet the surfaces of both the NiCuZn ferrite and the CaTiO_3_/BaTiO_3_ particles, and can effectively dissolve and transport ions, facilitating densification through liquid-phase sintering. However, due to the high melting point, CaTiO_3_/BaTiO_3_ particles are effectively pinned by the glassy phase at the grain boundaries. This creates a pinning effect that powerfully inhibits the growth of the ferrite grains, resulting in the significantly reduced grain size with the increasing CaTiO_3_/BaTiO_3_ contents as shown in the SEM images (Figure 3 and Figure 4).

**Figure 5:**
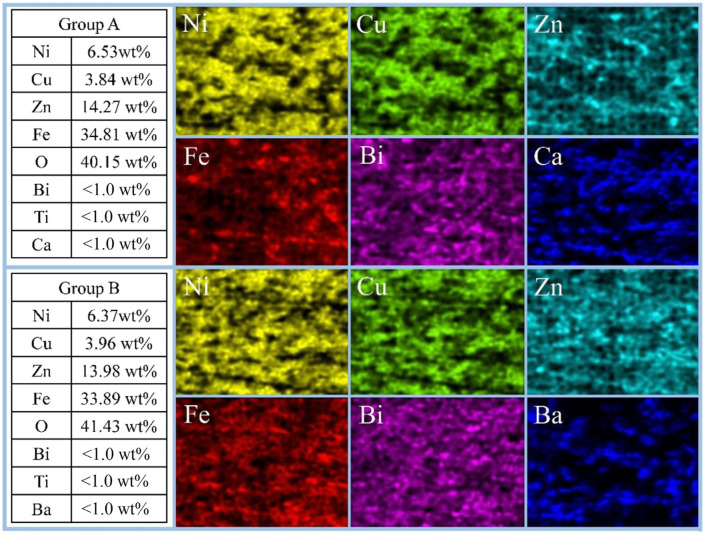
EDS elemental mapping of NiCuZn ferrites with the composite additive at x = 0.05 wt%. The distribution of characteristic elements (Ni, Cu, Zn, Fe, and Bi) and the respective additive elements (Ca for Group A, Ba for Group B) confirms their homogeneous distribution within the ferrite matrix, indicating effective compositing.

Figure 5 further presents the EDS surface distribution images of the typical elements, including Ni, Cu, Zn, Fe, Bi, Ca, and Ba in the NiCuZn samples with added 3.0 wt% BMLS and 0.05 wt% CaTiO_3_ or BaTiO_3_. It can be seen that all characteristic elements were evenly distributed in the measured area. Ca and Ba elements appeared in the as-prepared NiCuZn samples in Group A and Group B, respectively, indicating that the additive was effectively composited into the NiCuZn ferrites, which played a significant role in the modification of the microstructure and magnetic properties.

**Figure 6:**
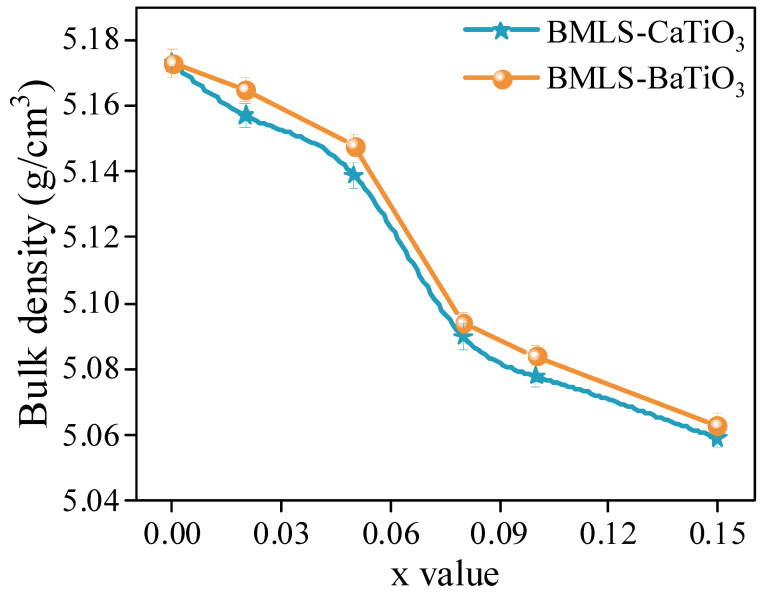
Variation in the bulk density of NiCuZn ferrites as a function of the MTiO_3_ content (x wt%). The composition follows the formula: (97 − x) wt% NiCuZnFe_1.98_O_4_ − 3 wt% BMLS glass − x wt% MTiO_3_ (M = Ca or Ba). A general decreasing trend in density is observed with increasing x for both Groups A and B.

Figure 6 displays the variation in the bulk density of NiCuZn ferrites. Both Group A and Group B samples exhibited a similar declining trend in bulk density as the concentration of either CaTiO_3_ or BaTiO_3_ was increased from 0.00 wt% to 0.15 wt%. The decrease occurred gradually at lower additive amounts; for instance, when x increased from 0.00 to 0.05, the density reduction was modest, with a reduction of 0.66% and 0.48% for Group A and Group B, respectively, relative to the NiCuZn with added BMLS samples. A more rapid reduction occurred around x = 0.08, where densities fell to approximately 5.09 g/cm^3^. By x = 0.15, the bulk densities in both groups reached their minimum values.

Generally, the bulk density of NiCuZn ferrites is closely related to their sintered densification behavior and final grain size. The small grain size and low densification degree indicate that NiCuZn ferrite has a low bulk density [11,26]. The SEM images of the microstructure displayed in Figure 3 and Figure 4 revealed that as the content of CaTiO_3_ or BaTiO_3_ increased, the effect of crystal growth inhibition became more pronounced, resulting in a gradual reduction in grain size and densification of the NiCuZn ferrite. In particular, when x = 0.08 and 0.15, the grain size of the NiCuZn samples decreased sharply. Therefore, corresponding to the results in Figure 6, the bulk density of the NiCuZn sample in group A and group B both showed a rapid decrease when x = 0.08 and 0.15. Moreover, it was also found that the bulk density of NiCuZn containing BMLS-CaTiO_3_ was lower than that of NiCuZn containing BMLS-BaTiO_3_ when the additive content was the same. This may be attributed to the density difference between CaTiO_3_ and BaTiO_3_. Since CaTiO_3_ has a lower density (4.1 g/cm^3^) than BaTiO_3_ (6.08 g/cm^3^), when the additive content was the same, CaTiO_3_ could occupy larger spatial positions and exhibited a more significant effect of crystal growth inhibition, resulting in more grain boundaries formed and smaller grains generated than BaTiO_3_, whereby the degree of densification decreased sharply. As a consequence, the NiCuZn sample with added BMLS-CaTiO_3_ had a lower bulk density than the NiCuZn sample with added BMLS-BaTiO_3_ at the same additive content. Furthermore, a synergistic effect on the microstructure is evident when correlating the bulk density, lattice strain, and grain size. The decline in density, coupled with the increasing microstrain and decreasing grain size, consistently points towards the role of Ca/BaTiO_3_ additives in inhibiting densification and grain growth through a pinning mechanism, ultimately resulting in smaller grain sizes and higher porosity in the structure.

**Figure 7:**
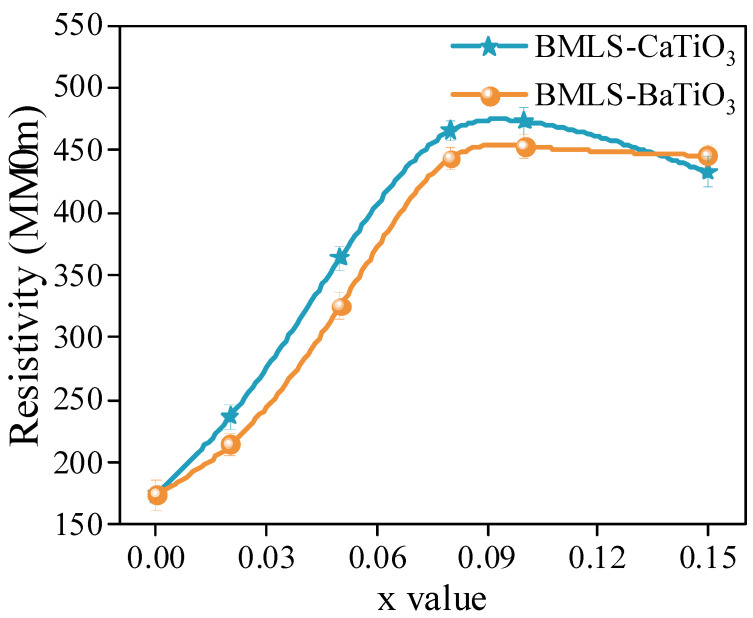
Dependence of the electrical resistivity of NiCuZn ferrites on the MTiO_3_ content (x wt%). The resistivity exhibits a non-monotonic behavior, initially increasing and then decreasing, with a maximum value achieved at x = 0.10 wt% for both groups.

Figure 7 illustrates the variation in resistivity of NiCuZn samples. Notably, the resistivity exhibited a non-monotonic behavior, initially rising and subsequently declining as the concentration of either CaTiO_3_ or BaTiO_3_ was increased. When x = 0.10, the NiCuZn samples in group A and group B obtained the maximum values, respectively. In general, a grain boundary has a much higher resistivity than a microcrystal. Thus, the resistivity of NiCuZn ferrite is primarily determined by the grain boundary [20,21,22]. The results in Figure 3 and Figure 4 indicated that the grain size of the NiCuZn samples gradually decreased as the additive content increased, indicating an obvious increase in the number of grain boundaries, thereby significantly improving the resistivity of the NiCuZn samples. Meanwhile, due to the relatively high melting point and high resistivity, CaTiO_3_ or BaTiO_3_ could be enriched at the grain boundary during the sintering process, which could further increase the resistivity of the NiCuZn samples [14,20,21,22]. Therefore, the resistivity of the NiCuZn samples showed an increasing variation trend with the increasing additive content. However, when the content of additives reached 0.15 wt%, excessive addition of CaTiO_3_ or BaTiO_3_ not only reduced the grain size, but also increased the porosity of the NiCuZn ferrites, which could significantly decrease the densification degree and the number of grain boundaries [10]. Therefore, a reduction in the resistivity was observed when x = 0.15. In addition, it was also found that NiCuZn containing BMLS-CaTiO_3_ exhibited a higher resistivity than NiCuZn containing BMLS-BaTiO_3_ when the x value was the same. This finding may be related to the fact that CaTiO_3_ contributed to a more significant effect of crystal growth inhibition to increase the number of grain boundaries and reduce the grain size (analyzed and confirmed by Figure 3, Figure 4, and Figure 6), thereby greatly improving the resistivity.

**Figure 8:**
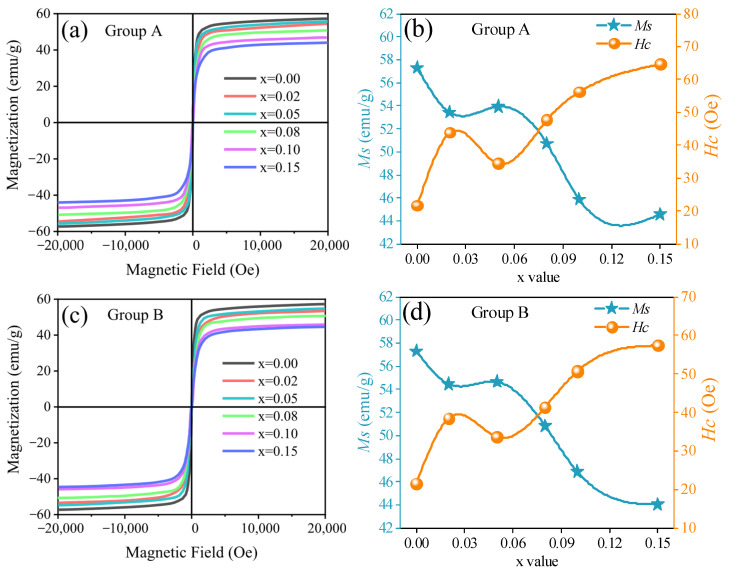
Magnetic properties of NiCuZn ferrites with added BMLS-CaTiO_3_/BaTiO_3_. (**a**,**c**) initial magnetization curves measured at room temperature. (**b**,**d**) Variation in saturation magnetization (*Ms*, in emu/g) and intrinsic coercivity (*Hc*, in Oe) as a function of the MTiO_3_ content (x wt%).

Figure 8 displays the initial magnetization curves and the variation trends of saturation magnetization (*Ms*) and intrinsic coercivity (*Hc*) for NiCuZn samples with added BMLS-CaTiO_3_/BaTiO_3_. As shown in Figure 8a,c, all samples exhibit typical ferrimagnetic hysteresis behavior under an applied magnetic field. Both figures indicate that the incorporation of either CaTiO_3_ or BaTiO_3_ significantly influences the saturation magnetization of the NiCuZn ferrites. The corresponding values of *Ms* and *Hc* for samples in groups A and B are quantitatively provided in Figure 8b,d, respectively. It can be seen that with the content of CaTiO_3_ or BaTiO_3_ increased, the *Ms* of the NiCuZn samples in two groups both decreased at first, then increased slightly, and finally decreased rapidly again. However, the *Hc* of the two kinds of NiCuZn samples exhibited the exact opposite variation trend. When x = 0.05, the *Ms* of NiCuZn samples in group A and Group B reached the values of 53.93 emu/g and 54.65 emu/g, respectively, which were reduced by 5.88% and 4.62% as compared with the *Ms* value when x = 0.00. At the same time, the *Hc* of the NiCuZn samples in two groups also exhibited a relatively low value of 34.4 Oe, and 33.7 Oe, respectively.

It has been established that the *Ms* and *Hc* are closely linked to the composition, density, and uniformity of grain size [15,27]. As evidenced by the XRD patterns in Figure 2 and the EDS results in Figure 5, the primary phase composition of the NiCuZn samples remained consistent. Therefore, the variations in *Ms* and *Hc* were mainly influenced by differences in density and grain size distribution. Generally, NiCuZn ferrites possessing high density and homogeneous grain morphology demonstrate superior magnetic performance, characterized by high *Ms* and low *Hc* [15,27]. However, as shown in Figure 3, Figure 4 and Figure 6, the incorporation of CaTiO_3_ or BaTiO_3_ led to a reduction in both grain size and density of the NiCuZn ferrites. Consequently, as the value of x increased from 0.00 to 0.15, *Ms* displayed a declining trend, while *Hc* progressively increased. However, when further comparing the *Ms* and *Hc* between x = 0.02 and x = 0.05, it was found that 0.05 wt% CaTiO_3_ or BaTiO_3_ samples exhibited increased magnetic properties than 0.02 wt% CaTiO_3_ or BaTiO_3_ samples. The microstructure of the NiCuZn samples is shown in Figure 3b and Figure 4b and demonstrates that although the grain size was slightly reduced as compared to that shown in Figure 3a and Figure 4a, the uniformity of the grain size was greatly enhanced when x = 0.05. Here, when x = 0.05, the positive influence induced by the enhanced grain size uniformity made a greater impact on the *Ms* and *Hc* than the negative influence induced by the reduced density. As a consequence, NiCuZn samples containing 0.05 wt% CaTiO_3_ or BaTiO_3_ exhibited a higher *Ms* and a lower *Hc* than NiCuZn samples containing 0.02 wt% CaTiO_3_ or BaTiO_3_. When x exceeded 0.05, the *M*s decreased rapidly and *Hc* increased sharply, which may be attributed to that the excessive addition of CaTiO_3_ or BaTiO_3_ resulted in a rapid deterioration of the grain size uniformity and density. Furthermore, the evolution of *Hc* and *Ms* should also be interpreted in light of the crystallite size revealed by Rietveld refinement (Table 2) and the effective fraction of magnetic material in the composite. The continuous decrease in crystallite size (t) with increasing x contributes significantly to the observed increase in *Hc*. According to the single-domain particle model, finer crystallites enhance the pinning of magnetic domain walls, thereby increasing the *Hc* [28]. Meanwhile, the incorporation of non-magnetic CaTiO_3_ and BaTiO_3_ phases introduces a magnetic dilution effect, which directly reduces the overall saturation magnetization (*Ms*) of the composite. Therefore, the general declining trend of *Ms* is attributed not only to the reduced density and grain size but also to this inherent dilution caused by the composite additives.

**Figure 9:**
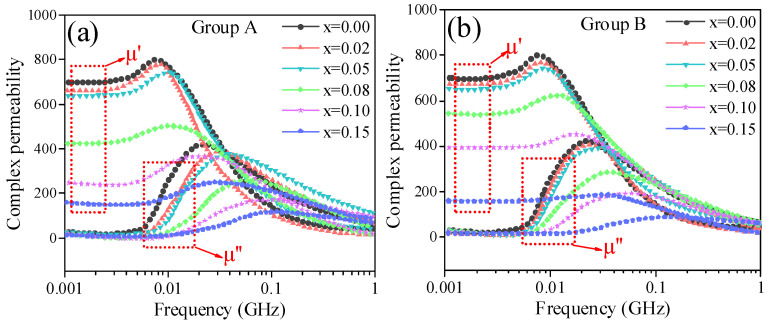
Frequency dependence of the complex permeability (real part μ′ and imaginary part μ″) for NiCuZn ferrites with varying MTiO_3_ content (x wt%). (**a**) NiCuZn ferrites with added BMLS-CaTiO_3_ and (**b**) NiCuZn ferrites with added BMLS-BaTiO_3_.The initial permeability (μ′ at low frequency) decreases progressively as x increases from 0.00 to 0.15 wt%.

Figure 9 gives the complex permeability spectra, including the real part of permeability (μ′) and imaginary part of permeability (μ″) of the NiCuZn samples in group A and group B. It was evident that the initial permeability of the NiCuZn samples in the two groups both decreased with the increasing content of CaTiO_3_ or BaTiO_3_. When x = 0.05, although the μ′ was reduced by the addition of CaTiO_3_ or BaTiO_3_, the NiCuZn samples in two groups still exhibited relatively high initial permeability of 636.95 and 651.55, respectively. When x increased from 0.05 to 0.15, the μ′ decreased rapidly with the increasing content of CaTiO_3_ or BaTiO_3_.

The complex permeability of ferrites is predominantly governed by two magnetization mechanisms: domain wall motion and domain spin rotation. However, in polycrystalline ferrites with fine grain size, the contribution from domain wall motion can be neglected [14,28,29]. As evidenced in Figure 3 and Figure 4, the grain size of all NiCuZn samples was below the single-domain critical size of 3 μm [30]. Consequently, the complex permeability of these samples was mainly attributable to spin rotation, which can be described by the following Equation (1) [31].

(1) $$\mu \approx 1+\chi_{spin}=1+\frac{4\pi M_{s}}{H_{d}+H_{A}}$$

where *Ms*, *Hd*, and *H_A_* respectively denote the ferrite’s saturation magnetization, demagnetization field, and magnetocrystalline anisotropic field. As shown in Figure 3, Figure 4, Figure 5, Figure 6, Figure 7 and Figure 8, when x increased from 0.00 to 0.05, *Ms* and density exhibit a slight decreasing tendency, and the uniformity of the grain size was greatly enhanced. Therefore, when x = 0.05, the NiCuZn samples in two groups still exhibited relatively high initial permeability of 636.95 and 651.55, respectively. When x exceeded 0.05, all the NiCuZn samples exhibited a rapid decrease in permeability, which may be attributed to the excessive addition of CaTiO_3_ or BaTiO_3,_ resulting in a rapid deterioration of the grain size uniformity and densification. When added excessively, the diffusion of these non-magnetic CaTiO_3_ or BaTiO_3_ in NiCuZn ferrite could significantly reduce the magnetism and decrease the grain size uniformity and densification, thereby resulting in a reduction in the magnetic permeability [32,33,34].

**Figure 10:**
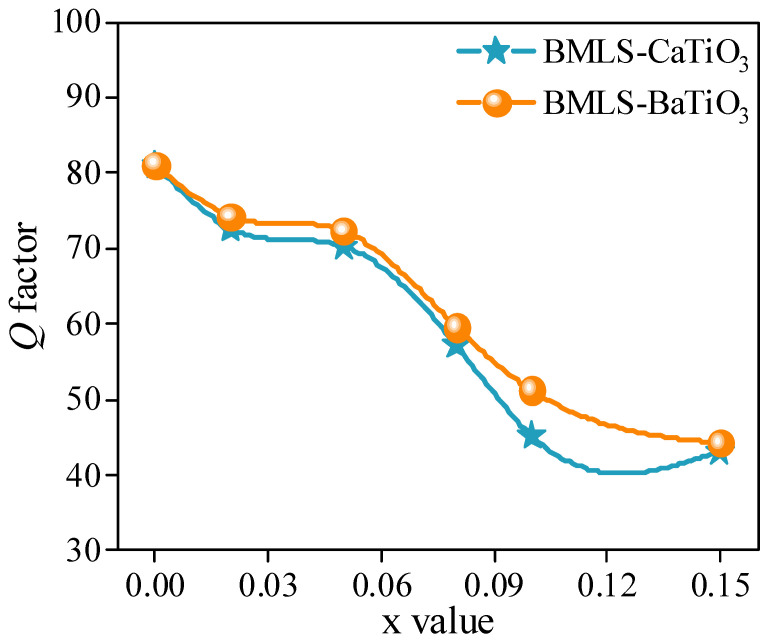
*Q* factor) of NiCuZn ferrites as a function of frequency, showing the effect of different MTiO_3_ contents (x wt%). The *Q* factor generally decreases with increasing x.

Figure 10 shows the variation in the *Q* factor of NiCuZn ferrites with added BMLS-CaTiO_3_/BaTiO_3_. Previous studies have indicated that a dense microstructure contributes to a high *Q* factor in ferrites [35,36]. Accordingly, the *Q* factor and density (Figure 6) followed a similar trend: both decreased gradually as x increased from 0.00 to 0.05. With a further increase in x from 0.05 to 0.15, the *Q* factor decreases sharply, which can be attributed to the pronounced decrease in density and densification.

**Figure 11:**
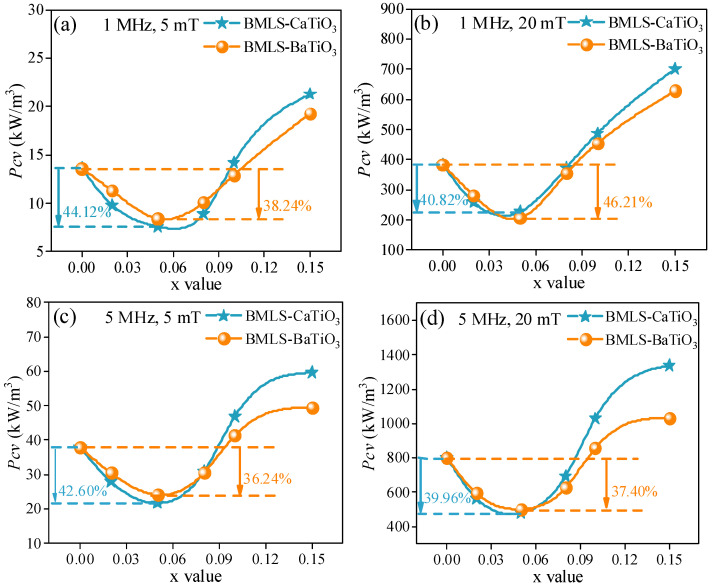
*Pcv* of NiCuZn ferrites under different (**a**,**c**) magnetic induction levels and (**b**,**d**) frequencies. The power loss for both Groups A and B first decreases, reaching a minimum at x = 0.05 wt%, and then increases with further increase in MTiO_3_ content (x).

Figure 11 illustrates the power loss behavior of NiCuZn samples containing BMLS-CaTiO_3_/BaTiO_3_ under various magnetic induction levels and frequencies. As the x increased from 0.00 to 0.15, all samples showed a consistent trend in power loss: an initial decrease followed by a sharp rise. Despite the slight reduction in complex permeability caused by CaTiO_3_/BaTiO_3_ incorporation (as indicated in Figure 9), the power loss properties were markedly enhanced across all frequencies and induction conditions. Notably, at x = 0.05, the samples achieved the minimum *Pcv* (total power loss) values, reflecting a reduction of more than 35.00% compared to the sample (x = 0.00). In general, the total power loss can be expressed by Equation (2), where *Ph*, *Pe*, and *Pr* denote hysteresis loss, eddy current loss, and residual loss, respectively [15,16].

*P*_*cv*_ = *P*_*h*_^+^
*P*_*e*_^+^
*P*_*r*_ (2)

According to the previous studies, the *Pr* (residual loss) was greatly affected by the Fe^2+^ content [14,16,37,38]. However, in this work, CaTiO_3_ and BaTiO_3_ both have a high melting point (over 1500 °C), which was much higher than the sintering temperature of 925 °C. These high melting point additives were difficult to melt and would not undergo chemical reactions throughout the process of sintering. The addition of CaTiO_3_ or BaTiO_3_ will not significantly change the Fe^2+^ content, and the *Pr* will not show an obvious change [20]. As a consequence, the *Pcv* was mainly determined by the *Ph* (hysteresis loss) and *Pe* (eddy current loss). The related studies showed that the *Pe* is inversely proportional to the resistivity and *Ph* is proportional to 1/*μ_i_*^3^ [14,15,16,19,39,40]. When x increased from 0.00 to 0.05, the resistivity of the NiCuZn samples displayed in Figure 7 was increased, and the complex permeability of the NiCuZn samples displayed in Figure 9 was slightly decreased, but all the samples still had a relatively high complex permeability (over 600). Thus, with the content of CaTiO_3_ or BaTiO_3_ increased from 0.00 wt% to 0.05 wt%, the decreased *Pe* made a more significant impact on the total power loss than the increased *Ph*. Therefore, the *Pcv* value was decreased with the x value increasing from 0.00 to 0.05, and reached the lowest value when x = 0.05. When x exceeded 0.05, the Ph was significantly increased because of the rapid reduction in complex permeability. As a consequence, although the resistivity still increased slowly, the *Pcv* was increased when x increased from 0.08 to 0.15.

In addition, when further comparing the effect on the power loss between CaTiO_3_ and BaTiO_3_, it can be found that NiCuZn samples containing BMLS-CaTiO_3_ exhibited a low power loss when the x value was between 0.00 and 0.05, whereas NiCuZn samples containing BMLS-BaTiO_3_ exhibited a low power loss when the x value exceeded 0.08. The complex permeability of NiCuZn samples under two compositing modes exhibited a relatively high value when the x value was between 0.00 and 0.05. Therefore, the difference in power loss of the samples was mainly determined by the *Pe*. As shown in Figure 7, NiCuZn samples with added BMLS-CaTiO_3_ exhibited a higher resistivity than NiCuZn samples with added BMLS-BaTiO_3_. Thus, NiCuZn samples containing BMLS-CaTiO_3_ obtained a lower *Pe*, and exhibited a relatively low *Pcv* value accordingly when x was between 0.00 and 0.05. When x exceeded 0.08, Figure 9 showed that the addition of CaTiO_3_ resulted in a faster decrease in the complex permeability than BaTiO_3_, contributing to a significant increase in *Ph*. Therefore, when x exceeded 0.08, NiCuZn samples containing BMLS-BaTiO_3_ exhibited a relatively low power loss.

## 4. Conclusions

In this study, to achieve NiCuZn ferrites with excellent magnetic performance and low power loss, composite additives comprising BMLS-CaTiO_3_ and BMLS-BaTiO_3_ were introduced to modify the properties of the ferrites sintered at a low temperature of 925 °C. The effects of BMLS-CaTiO_3_ (Group A) and BMLS-BaTiO_3_ (Group B) on the phase composition, density, resistivity, microstructure, magnetic properties, and power loss of NiCuZn ferrites were systematically investigated, and the underlying mechanisms were thoroughly discussed. The main findings are as follows:

(1) NiCuZn ferrites incorporating BMLS-CaTiO_3_/BaTiO_3_ as composite additives were successfully prepared at a low sintering temperature of 925 °C, and all samples exhibited a pure spinel phase structure.
(2) The microstructure evolution with the CaTiO_3_/BaTiO_3_ content (x) was systematically revealed. As x increased from 0.00 to 0.15 wt%, the average grain size exhibited a continuous reduction. Notably, the grain size uniformity initially improved, reaching an optimum at x = 0.05 wt%, before deteriorating at higher concentrations (x ≥ 0.08 wt%) due to excessive inhibition of grain growth.
(3) As the content of CaTiO_3_ or BaTiO_3_ increased, the density, complex permeability, and *Q* factor consistently decreased. The resistivity, however, showed a non-monotonic dependence on x, reaching a maximum at x = 0.10 wt%. This trend is attributed to two competing effects: the initial increase is due to the increased number of high-resistivity grain boundaries and the segregation of insulating CaTiO_3_/BaTiO_3_ phases, which impede electron transport. The subsequent decrease at x = 0.15 wt% is likely caused by the excessive porosity and degraded densification, which can create defective paths and outweigh the benefits of grain refinement.
(4) With increasing CaTiO_3_/BaTiO_3_ content, *Ms* and permeability followed a general declining trend, while the *Hc* exhibited a continuous increase. The sample at x = 0.05 wt% represents an optimal compromise within this trend (*Ms* > 53.9 emu/g, permeability > 636), where the positive effect of enhanced grain uniformity partially counteracts the negative impacts of magnetic dilution, crystallite size reduction, and density decrease.
(5) As the content of CaTiO_3_ or BaTiO_3_ increased, the total power loss of the samples first decreased and then increased. The lowest power loss was achieved at x = 0.05, with a reduction exceeding 35.00% across various magnetic induction levels and frequencies. This reason is mainly due to increased resistivity, which significantly lowers eddy current loss. However, when x exceeds 0.05, the sharp decrease in complex permeability contributes to a substantial increase in hysteresis loss, ultimately raising the total power loss despite the continued increase in resistivity.
(6) This study demonstrates that the microstructure and electromagnetic properties of low-temperature-sintered NiCuZn ferrites can be effectively tailored by the BMLS-Ca/BaTiO_3_ composite additives. The evolution of these parameters with x offers a clear guideline for material selection based on application needs: compositions with x ≤ 0.05 wt% are optimal for applications requiring high permeability and low power loss (e.g., high-efficiency inductors), whereas compositions with higher x values (e.g., 0.10 wt%), which exhibit peak resistivity, might be better suited for RF and microwave device applications.

## Figures and Tables

**Table 1:** Typical properties of CaTiO_3_ and BaTiO_3_.

Item	Density (g/cm^3^, 25 °C)	Melting Point (°C)	Molecular Weight
CaTiO_3_	4.1	1975	135.94
BaTiO_3_	6.08	1625	233.19

**Table 2:** Variation of lattice constant, strain (ε), and crystallite size (t) of the NiCuZn ferrites with BMLS-Ca/BaTiO3.

Additive contents		0.02	0.05	0.08	0.10	0.15
CaTiO_3_	a (Å)	8.4148	8.4143	8.4129	8.4113	8.4091
	ε (×10^−4^)	1.19	2.85	4.75	5.94	8.37
	t (nm)	47.38	44.63	42.36	38.67	28.28
BaTiO_3_	a (Å)	8.4149	8.4145	8.4134	8.4121	8.4095
	ε (×10^−4^)	2.26	3.30	4.95	6.49	9.50
	t (nm)	48.07	45.03	42.16	39.68	30.38

## Data Availability

The original contributions presented in the study are included in the article, further inquiries can be directed to the corresponding authors.
